# The complete chloroplast genome sequence of *Bambusa vulgaris* cv. Wamin

**DOI:** 10.1080/23802359.2021.1927870

**Published:** 2021-05-21

**Authors:** Zhiwen Deng, Liting Huang, Meng Zhang, Muhammad Waqqas Khan Tarin, Tianyou He, Linyan Chen, Jundong Rong, Liguang Chen, Yushan Zheng

**Affiliations:** a College of Forestry, Fujian Agriculture and Forestry University, Fuzhou, Fujian, China; b College of Landscape Architecture, Fujian Agriculture and Forestry University, Fuzhou, Fujian, China

**Keywords:** *Bambusa vulgaris* cv. Wamin, plastid genome, phylogenetic analysis, Bambusoideae

## Abstract

*Bambusa vulgaris* cv. Wamin is an attractive ornamental bamboo species of southern China. It has large swollen internodes and weeping culms, and it has considerable economic importance. In the present study, we sequenced the complete chloroplast genome of *B. vulgaris* cv. Wamin and reported it for the first time. The genome was 139,528 bp in total length, including a large single-copy (LSC) region of 83,038 bp, a small single-copy (SSC) region of 12,893 bp, and a pair of invert repeats (IR) regions of 21,799 bp. Plastid genome contained 138 genes, 82 protein-coding genes, 38 tRNA genes, and 8 rRNA genes. The overall GC content of the genome was 38.9%. The phylogenetic analysis based on the complete chloroplast genome reveals that *B. vulgaris* cv. Wamin is closely related to *Bambusa teres*. This research strengthens the genetic information of both the *B. vulgaris* cv. Wamin and the phylogenetic analyses of Gramineae.

*Bambusa vulgaris* cv. Wamin is a cultivated bamboo species (genus Leucaena; family Gramineae), a shrub or tree-like bamboo plant found in southern China and the provinces of Zhejiang, Fujian, and Taiwan, where it has been introduced as an ornamental plant (http://www.iplant.cn/). It is very important to acquire *B. vulgaris* cv. Wamin genetic and genomic information and also to pursue the study of its phylogenetic significance and preservation. The chloroplast genome has a maternal history and a preserved structure that has been used to investigate plant developmental and phylogenetic relationships (Wang et al. 2018). Therefore, we identified the complete chloroplast genome of *B. vulgaris* cv. Wamin by next-generation sequencing (NGS) technology. In addition, a phylogenetic analysis was also presented of this species.

Fresh leaves tissues of *B. vulgaris* cv. Wamin were obtained from the Bamboo Garden of Fujian Agriculture and Forestry University, Fuzhou, Fujian province, China (119°14′6″E, 26°5′7″N). Collected samples were then immediately dried into the silica gel. The specimens were stored in the Herbarium of College of Forestry, Fujian Agriculture and Forestry University (specimen code #101306). DNA was isolated from fresh leaf tissues, while its quantification was confirmed using Agarose gel electrophoresis and Nanodrop concentration, by 500 bp randomly interrupted by the Covaris ultrasonic breaker for library construction. About 2.0 GB of raw data was produced by 150 bp paired-end read lengths. The Illumina High-throughput sequencing platform (HiSeq2500) data was processed by the NOVOPlasty script (Dierckxsens et al. 2017). The complete plastid genome of *B. vulgaris* (GeneBank accession: NC 050780) as comparison and plastid genome of *B. vulgaris* cv. Wamin was assembled by GetOrganelle pipe-line (https://github.com/Kinggerm/GetOrganelle), the plastid-like reads were obtained, and the readings were examined and modified by Bandage (Wick et al. 2015). The chloroplast genome database was compiled based on Geneious v 11.1.5 (Biomatters Ltd., Auckland, New Zealand) reference (Kearse et al. 2012).

The final complete chloroplast genome sequence of *B. vulgaris* cv. Wamin was submitted to GenBank under accession number MW544010. Raw reads were deposited in the GenBank Sequence Read Archive (SRA PRJNA698784). The complete plastid genome sequence of *B. vulgaris* cv. Wamin was 139,528 bp in length, with a large single-copy (LSC) region of 83,038 bp, a small single-copy (SSC) region of 12,893 bp, and a pair of inverted repeats (IR) regions of 21,799 bp. The complete chloroplast genome comprised 138 genes, including 82 protein-coding genes, 38 tRNA genes, and 8 rRNA genes. The complete genome GC content was 38.9%. To reveal the phylogenetic position of *B. vulgaris* cv. Wamin with other members of Bambusa, we performed a phylogenetic analysis based on 15 complete chloroplast genomes of Bambusa, and one taxa (*Dendrocalamus barbatus*) as outgroups. All of them were downloaded from NCBI GenBank. The sequences were aligned by MAFFT v7.307 (Katoh and Standley 2013), and the phylogenetic tree was constructed by RAxML (Stamatakis 2014) with 1000 bootstrap replicates. The GTRGAMMA model was used in the ML analysis. Phylogenetic analysis findings clearly confirm the close association between *B. vulgaris* cv. Wamin and *Bambusa teres* (Figure 1).

**Figure 1.**
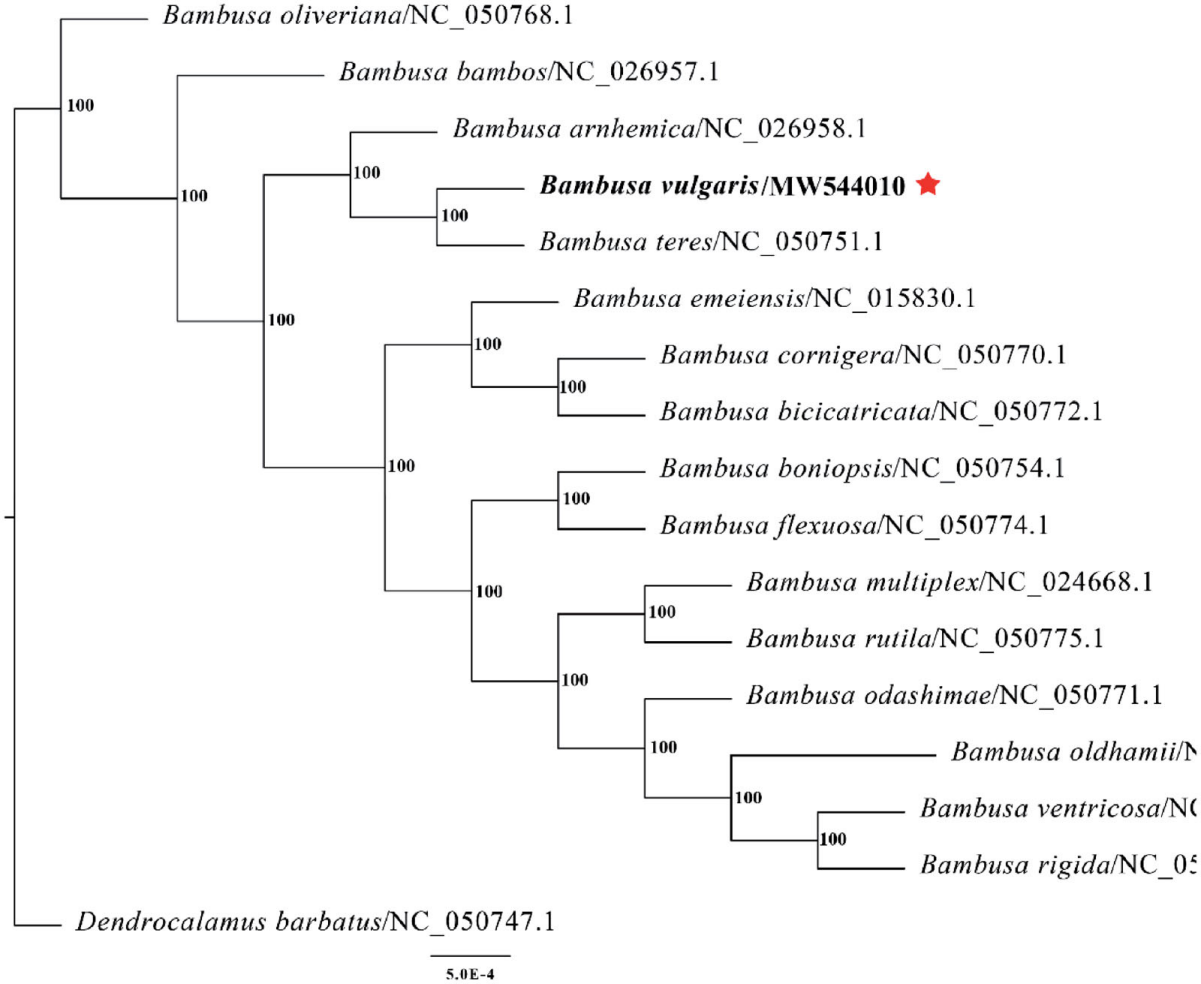
Maximum-likelihood phylogenetic tree based on complete chloroplast genomes. Numbers close to each node are bootstrap support values.

## Data Availability

The data that support the findings of this study are openly available in GeneBank of NCBI at https://www.ncbi.nlm.nih.gov/ with the following accession number MW544010. All high-throughput sequencing data files are available from the GenBank Sequence Read Archive (SRA) accession number: PRJNA698784.

## References

[CIT0402] Dierckxsens N, Mardulyn P, Smits G. 2017. Novoplasty: De novo assembly of organelle genomes from whole genome DNA. Nucleic Acids Res. 45:e18. 10.1093/nar/gkw955 PMC5389512 28204566

[CIT0001] Katoh K, Standley DM. 2013. MAFFT multiple sequence alignment software version 7: improvements in performance and usability. Mol Biol Evol. 30(4):772–780. 23329690 10.1093/molbev/mst010 PMC3603318

[CIT0002] Kearse M, Moir R, Wilson A, Stones-Havas S, Cheung M, Sturrock S, Buxton S, Cooper A, Markowitz S, Duran C, et al. 2012. Geneious basic: an integrated and extendable desktop software platform for the organization and analysis of sequence data. Bioinformatics. 28(12):1647–1649. 22543367 10.1093/bioinformatics/bts199 PMC3371832

[CIT0003] Stamatakis A. 2014. RAxML version 8: a tool for phylogenetic analysis and post-analysis of large phylogenies. Bioinformatics. 30(9):1312–1313. 24451623 10.1093/bioinformatics/btu033 PMC3998144

[CIT0004] Wang J, Li C, Yan C, Zhao X, Shan S. 2018. A comparative analysis of the complete chloroplast genome sequences of four peanut botanical varieties. PeerJ. 6:e5349. 30083466 10.7717/peerj.5349 PMC6074784

[CIT0005] Wick RR, Schultz MB, Zobel J, Holt KE. 2015. Bandage: interactive visualization of de novo genome assemblies. Bioinformatics. 31(20):3350–3352. 26099265 10.1093/bioinformatics/btv383 PMC4595904

